# Synthetic Augmented Suture Anchor Reconstruction for a Complete Traumatic Distal Triceps Tendon Rupture in a Male Professional Bodybuilder with Postoperative Biomechanical Assessment

**DOI:** 10.1155/2014/962930

**Published:** 2014-02-16

**Authors:** Maria-Elissavet Nikolaidou, Ingo J. Banke, Thomas Laios, Konstantinos Petsogiannis, Anastasios Mourikis

**Affiliations:** ^1^Sport Biomechanics Laboratory, Department of Physical Education and Sport Science, National and Kapodistrian University of Athens, Ethnikis Antistasis 41, Dafne, 172-37 Athens, Greece; ^2^Clinic of Orthopedics and Sports Orthopedics, Rechts der Isar Hospital, Technical University of Munich, Ismaninger Street 22, 81675 Munich, Germany; ^3^Department of Orthopaedics, “Laiko” General Hospital, Agiou Thoma Street 17, 11527 Athens, Greece

## Abstract

Bodybuilding is a high-risk sport for distal triceps tendon ruptures. Management, especially in high-demanding athletes, is operative with suture anchor refixation technique being frequently used. However, the rate of rerupture is high due to underlying poor tendon quality. Thus, additional augmentation could be useful. This case report presents a reconstruction technique for a complete traumatic distal triceps tendon rupture in a bodybuilder with postoperative biomechanical assessment. A 28-year-old male professional bodybuilder was treated with a synthetic augmented suture anchor reconstruction for a complete triceps tendon rupture of his right dominant elbow. Postoperative biomechanical assessment included isokinetic elbow strength and endurance testing by using multiple angular velocities to simulate the “off-season” and “precompetition” phases of training. Eighteen months postoperatively and after full return to training, the biomechanical assessment indicated that the strength and endurance of the operated elbow joint was fully restored with even higher ratings compared to the contralateral healthy arm. The described reconstruction technique can be considered as an advisable option in high-performance athletes with underlying poor tendon quality due to high tensile strength and lack of donor site morbidity, thus enabling them to restore preinjury status and achieve safe return to sports.

## 1. Introduction

Bodybuilders, along with weight-/powerlifters and football players, are among the most susceptible for distal triceps tendon ruptures [1–4]. Even though these injuries represent the 0.8% of all upper extremities tendon injuries in athletes [5], the risk of exhibiting a distal triceps tendon rupture in bodybuilding is particularly high [6–8]. Predisposing factors are anabolic steroid use [8–10], local steroid injection, and chronic tendinopathy [1, 11]. Anabolic steroid use has been shown to have a detrimental ultrastructural effect on the volume and quality of tendon collagen fibrils [9]. Recent studies report on the extensive damage to the musculotendinous unit after long-term steroid abuse [11] and intramuscular oil injections [12]. Furthermore, repetitive eccentric exercise, as the high volume resistance training employed in bodybuilding [13], is considered a predisposing factor due to an elevated risk of chronic tendinopathy.

Management in top-level athletes is operative and well established with good mid- to long-term results and return to sports [3, 14]. Many techniques exist such as the currently favored anatomical suture anchor refixation [4, 5, 15]. However, in competitive bodybuilding, only very few reports exist regarding triceps tendon rupture and operative reconstruction [2, 6–8, 16]. The outcome of primary tendon repair depends on tendon quality and injury chronicity [11, 17]. Rerupture is the most frequent complication [5, 18]. Bodybuilders exhibit a particular high risk of rerupture due to predominantly poor tendon quality [10], challenging rehabilitation strategy, and (too) early return to high-load training in order to minimize loss of muscle volume [6, 16]. In top-level athletes [3, 6, 18] additional autograft, allograft, or even synthetic augmentation may be promising resulting in good functional outcome [4, 19]. Using tendon autograft augmentation in bodybuilders might be disadvantageous due to the possible generalized tendinopathy and donor site muscular disfiguring leading to disappointing cosmetics [13]. However, to the best of our knowledge, no case with synthetic augmentation is described in high-demand patients as bodybuilders.

The literature is also lacking information on the performance outcome of the repair strength in high-demand patients. During the rehabilitation phase, valid and objective data other than subjective satisfaction and function reports are needed to assess whether sufficient strength and endurance levels have been achieved determining return to the athlete's preinjury activities [17]. Up to now, testing protocols with a single isokinetic velocity evaluating the strength and endurance recovery outcome are employed [20, 21]. However, training practices of bodybuilders differ. During the off-season period, moderate-to-high loads and moving the weight in a controlled, often slow (>2-3 s) fashion are used. During the precompetition phase, with the focus on retaining muscle mass, a number of repetitions are increased using lighter loads and moving the weight faster [13, 22]. Thus, a single isokinetic velocity test may only provide vague prediction of the capability of full return to sports possibly increasing the risk of rerupture. Rather, extensive isokinetic testing would be more meaningful to indicate the extent of strength repair and ensure safe return to training and competition.

This case report describes the synthetic augmented suture anchor reconstruction technique used to treat a complete traumatic distal triceps tendon rupture in a male professional bodybuilder with underlying poor tendon quality and the postoperative biomechanical assessment.

## 2. Case Presentation

### 2.1. Patient History and Diagnosis

**Figure 1:**
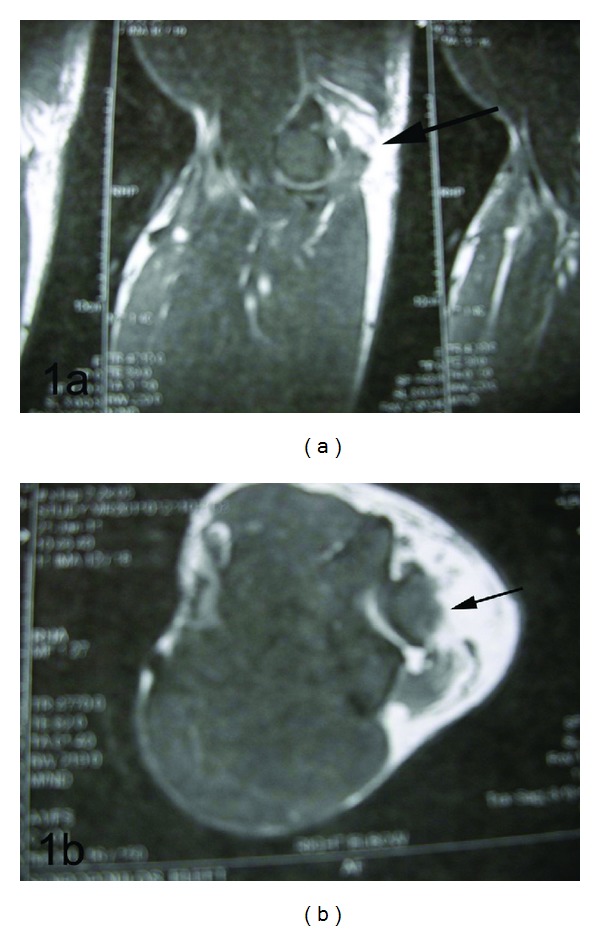
Preoperative MRI (scanned from hard copy) with arrows indicating the complete distal triceps tendon rupture at its insertion at the olecranon in a sagittal (a) and transverse (b) view.

A 28-year-old male professional bodybuilder competing the national championships with 98.7 kg/1.82 cm suffered an acute triceps rupture of his right dominant elbow while performing high-load close grip triceps bench press. Clinically a painful gap at the distal triceps tendon insertion was evident. The patient was unable to do active forearm extensions. MRI performed four days after trauma confirmed the diagnosis of a complete distal triceps tendon avulsion (Figure 1). No previous history of tendon rupture was reported. The patient confirmed long-term (anabolic) steroid abuse.

### 2.2. Patient Treatment: Surgical Reconstruction Technique

**Figure 2:**
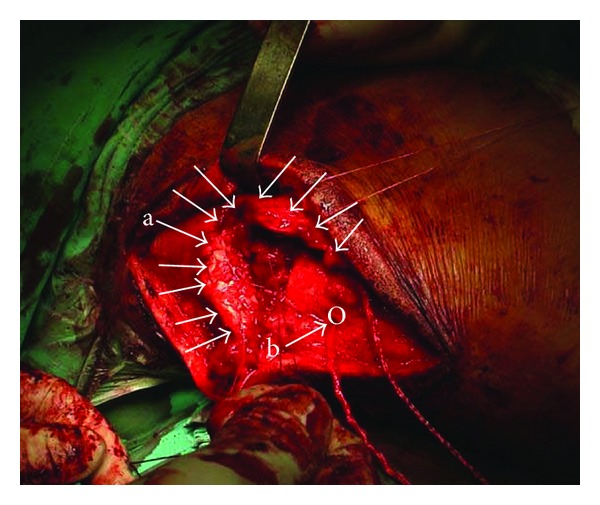
Intraoperative aspect indicating (a) the ruptured and retracted distal triceps tendon and (b) the synthetic tendon allograft already positioned through the olecranon (O). Suture anchors were placed afterwards.

After posterior longitudinal approach of the elbow, a complete distal triceps tendon rupture at the insertion site could be identified. Significant tendon debridement up to healthy tendon tissue had to be performed due to overall very poor tendon quality. Mobilisation of the tendon to its debrided insertion at the olecranon was only possible applying massive tension. Due to tissue poor quality and the need of high tension forces to reattach tendon to bone, a synthetic additional augmentation was employed in this acute repair. In detail, a double-stranded polyester-polyester material with failure strength of 2300N (Synthetic Tendon Ligament “TOW,” Surgicraft Ltd., Redditch, UK) braided together in a “zig-zag” configuration up to the level of the musculotendinous junction was woven through the tendon stump. Via a transverse drill hole through the olecranon the construct could be tightened, approximating the synthetic augmented distal triceps tendon close to the olecranon insertion (Figure 2). Then tensile free full surface reattachment and fine adaption of the tendon to the bone with three titanium suture screw anchors (Orthomed, St. Jeannet, France) were performed. Intraoperative assessment in full flexion and extension revealed a stable refixation without anchor displacement. Histopathological examination of the tendon's stump debrided material showed extensive degeneration including fibrinoid necrosis, fibrosis, and inflammation.

Postoperatively, the joint was protected with a functional elbow brace (Innovator-X, AliMed, Dedham, USA). Rehabilitation consisted of ROM exercises with a limitation of 90° of passive flexion for the first 6 weeks. At the 12th postoperative week, ROM was 0°–120°. Strengthening exercises were applied. Six months after injury, the bodybuilder returned to his preinjury “off-season” level and at eight months he started following an intense preparation program in order to participate in national championships.

### 2.3. Postoperative Biomechanical Assessment

Strength and endurance of the distal triceps tendon repair was tested 18 months postoperatively using isokinetic dynamometry (Cybex II+, New York, USA). As the triceps tendon rupture occurred during concentric elbow extensor contraction, concentric isokinetic testing was chosen to rationally evaluate the patient after triceps repair in a similar condition to the one during his trauma. However, eccentric testing was not our main focus and thus not performed.

Written informed consent was obtained from the patient. The study was in accordance with ethical standards for research on human subjects [23] and was approved by the Ethics Committee of the School of Physical Education and Sport Science, University of Athens, Greece.

The biomechanical evaluation was targeted to simulate exercise conditions used during the “off-season” and the “precompetition” training phases of bodybuilders [13, 22] by using two slow and two faster angular speeds, respectively. Forearm rotation, even though not directly affected by the triceps brachii muscle, was also tested for a possible weakness due to compensatory overloading of the biceps brachii. The patient's left nondominant arm was used as the control side.

#### 2.3.1. Strength Assessment

Peak isokinetic strength testing was conducted for elbow extension-flexion at 30-45-90-180°/s and forearm pronation-supination at 90°/s and 180°/s modified as described [21]. Familiarization included 5–7 submaximal warm-up repetitions at a randomly selected velocity. After a 3 min rest, testing started with elbow extension-flexion followed by forearm pronation-supination. The bodybuilder was stabilized in supine position, his shoulder joint was horizontally abducted at 45°, and the dynamometer's axis was aligned with the humerus epicondyle, while the forearm was in neutral position. Elbow's ROM was set between 30° and 135° flexion. Starting in full elbow flexion, 3 trials of concentric extension-flexion were performed with 1 min rest between velocities.

Forearm pronation-supination testing was performed while sitting upright on the dynamometer, having the elbow flexed at 90° and the forearm firmly stabilized and modified as described [21]. Forearm's ROM was 0°–50° in both directions. Testing started in maximal forearm supination and was completed after 3 trials of concentric pronation-supination with 1 min rest between velocities. From the strength measurements, the trial with the higher peak torque values was selected.

#### 2.3.2. Endurance Assessment

Endurance of both arms was tested involving 25 continuous elbow extension-flexion movements at 90°/s. Testing position, stabilization, and elbow's ROM were identical as those used in elbow joint testing. Five submaximal warm-up repetitions at 90°/s preceded testing. From a total of 25 trials, the mean peak torque for trials 2 to 3 as compared to that for trials 23 to 24 was selected [21].

#### 2.3.3. Data Collection and Analysis

During measurements, testing order was randomized across velocities and side. The torque signal was sampled at 1,000 Hz using the MP100 acquisition system via AcqKnowledge software (Biopac Systems Inc., USA). From all selected trials, the mean peak torque over a 100 ms window was used in final analyses. To calculate possible difference in strength and endurance, a typical formula was used: *d* = ((PT_OPER_ − PT_CONTROL_/PT_OPER_)∗100%), where PT_OPER_ and PT_CONTROL_ are peak torque value for operated and control side, respectively. Positive values indicate that strength or endurance of operated side is higher than that of control side.

### 2.4. Biomechanical Assessment Outcome

Six months after injury, the bodybuilder returned to his preinjury “off-season” level and at 8 months he started following an intense preparation program for the national championships. 18 months postoperatively full, return to sports with symptom- and limitation-free maximum weight bearing could be observed. In order to control muscle volume before trauma and after performed triceps repair, the upper arm circumference of 20 cm and the forearm circumference of 10 cm in relation to the lateral/radial humeral epicondyle were investigated. Thereby, an upper arm circumference of 55 cm (dominant right side) and 53 cm (left side) before trauma and of 52 cm (dominant right side) and 53 cm (left side) 18 months postoperatively could be obtained. The forearm circumference measurement resulted in 38 cm (both sides) before trauma and 36.5 cm (dominant right side) and 38 cm (left side) 1.5 years after triceps repair.

Eighteen months postoperatively, peak isokinetic strength of the dominant operated arm was higher than that of the contralateral healthy arm for elbow extension by 13% to 23% and elbow flexion by 20% to 30% (Table 1). Peak forearm supination strength revealed a greater relative difference between operated and control side than pronation strength (Table 1).

Results for endurance testing at 90°/s speed showed a 16% higher endurance strength in elbow flexion and no relevant deficit in elbow extension endurance of the treated arm compared to the healthy contralateral control arm (Table 2).

## 3. Discussion

This case report describes the full return to sports in a professional bodybuilder 1.5 years after synthetic augmented suture anchor reconstruction for a complete traumatic rupture of his right distal triceps tendon underlying poor tendon quality.

Bodybuilding is considered a high-risk sport for distal triceps tendon rupture [1, 2, 4]. Training regimes of bodybuilders include several predisposing factors. Specifically, systematic anabolic steroid use [8, 9] as in our case is frequent in bodybuilding, either present as ergogenic aids supplementing their diet [11, 24], or as intramuscular injections for achieving muscle hypertrophy [10, 12]. Another factor is chronic degenerative tendinopathy, as in our case, which can be attributed to the repetitive high eccentric loading of the upper extremities [4, 22].

Several case reports exist describing various reconstruction techniques with suture anchor refixation being the most popular [4, 5, 15]. However, in high-demand professional bodybuilding, information is still limited [2, 6–8, 16] and operative treatment decisions are individualized based on the patient's injury history and functional status [4, 5, 17]. Overall, augmentation techniques using allografts seem to have a reasonable outcome in terms of durability and longevity [4, 19]. No comparative studies using augmentation techniques with allografts versus autografts have been published, so the efficiency of the augmentation technique used here cannot be fully evaluated. Our decision to use a synthetic augmentation instead of an autograft [4] was theoretically based on the consideration of a possible general tendinopathy present in bodybuilding and also on the necessity to preserve the bodybuilder's aesthetic muscular appearance by avoiding scars and possible muscular disfiguring at the donor site [13]. Employing an allograft (hamstring tendon) augmentation as proposed by many authors in such difficult acute tears resulting in moderate-to-good results [4] would have been a valuable alternative in our case. However practically it was not available for us due to European Ethical Standards of our country. The synthetic allograft's biomechanical features provide high tensile strength and elasticity during the functional range of elbow joint motion. Thus, it can create a musculotendinous unit able to tolerate the high-load training demands of bodybuilding and acts as a scaffold providing the ingrowth of the newly formed tendon collagen fibres.

To our knowledge, this is the first study addressing the postoperative performance outcome of a high-demand athlete after synthetic augmented reconstruction. Therefore an individualized protocol focusing on the patient's preinjury status and functional demands was designed. The biomechanical assessment protocol used multiple isokinetic velocities targeting at simulating bodybuilding training conditions [13, 22] in order to objectively review the strength of the reconstruction. Previous authors have used a single isokinetic velocity to test for the strength of repair in weightlifters with ruptured triceps [20, 21]. Other authors have only provided subjective data describing the extent of recovery [1, 2, 6–8, 18]. Only van Riet et al. [5] reviewed their results of reconstructions and found that the average peak isokinetic strength of the reconstructed side was 66% compared with the uninvolved one in 9 patients at an average of 42 months after surgery. Foulk and Galloway [14] showed that their 16-year-old football player had a 6% peak torque to body weight deficit in extension at 60°/s and 2.1% deficit at 120°/s, at 11 months after injury. Our findings show that, at 18 months after surgery, the bodybuilder's peak isokinetic strength and endurance of the operated dominant elbow could be fully restored and was higher than his control elbow. Forearm rotation, however not being directly affected by the triceps brachii muscle, was also tested for a possible weakness due to possibly compensatory overloading of the biceps brachii in poor triceps tendon quality. No weakness could be found in forearm pronation/supination.

In accordance, the bodybuilder had subjectively returned to his preinjury strength and endurance levels without remaining complaints or restrictions winning the national bodybuilding championship 2.5 years after surgery.

In conclusion, the described reconstruction technique with simultaneous additional synthetic suture anchor augmentation might be a valuable option in professional high-demand patients as bodybuilders or weightlifters with poor tendon quality and high cosmetic demands in order to restore their preinjury status and ensure safe return to full athletic activity. Taking into account the information presented here, (sports) orthopaedic- and traumatologic-orientated colleagues could find the present case report helpful upon selecting the appropriate treatment and rehabilitation rationale for high-demand patients with ruptured tendons.

## Figures and Tables

**Table 1:** Peak isokinetic strength (Nm) testing in elbow extension-flexion and forearm pronation-supination.

Angular velocity	Elbow extension			Elbow flexion		
	Operated side^a^	Control^b^	*d* (%)^c^	Operated side	Control	*d* (%)
30°/s	40.5	31.3	+22.6	57.0	40.6	+28.9
45°/s	40.2	35.0	+12.9	60.1	44.1	+26.7
90°/s	39.8	32.6	+18.0	58.5	40.8	+30.3
180°/s	33.4	34.1	−2.1	47.7	38.3	+19.7

	Forearm pronation			Forearm supination		

90°/s	9.1	5.6	+38.7	13.8	4.2	+69.7
180°/s	11.5	4.3	+62.3	6.9	3.4	+50.6

^a^Operated side: right dominant arm; ^b^control: left nondominant arm; ^c^ *d* (%): percentage difference between operated and control side.

**Table 2:** Elbow extension-flexion endurance (Nm) testing.

Angular velocity^a^	Elbow extension			Elbow flexion		
	Operated side^b^	Control^c^	*d* (%)^d^	Operated side^a^	Control^b^	*d* (%)^d^
Trials 2-3	33.6	26.0	+22.7	47.2	33.6	+28.8
Trials 23-24	33.5	34.1	−1.6	39.9	33.5	+16.0

^a^For endurance testing of the elbow joint, only the 90°/s angular velocity was used; ^b^operated side: right dominant arm; ^c^control: left nondominant arm; ^d^ *d* (%): percentage difference between operated and control side.
